# Supplementary material for: Altered microRNA expression correlates with reduced TLR2/4-dependent periodontal inflammation and bone resorption induced by polymicrobial infection
Source: Microbiol Spectr. 2025 Aug 14;13(10):e00160-25. doi: 10.1128/spectrum.00160-25 (PMC12502665; doi:10.1128/spectrum.00160-25)
Supplement: Supplemental figures — Fig. S1 to S3. [file spectrum.00160-25-s0001.docx]

**Altered microRNA Expression Correlates with Reduced TLR2/4-Dependent Periodontal Inflammation and Bone Resorption Induced by Polymicrobial Infection.**

Syam Jeepipalli**^1^**^†^, Parvathi G**^1^**, Ana Rafaela Luz Martins**^1^**, Eduardo Colella**^1^**, Sandhya R Nadakuditi**^1^**, Tushar Desaraju**^1^**, Ashitha Yada**^1^**, Jennifer Onime**^1^**, John Williams**^2^**, Indraneel Bhattacharyya^3^, Edward K. L. Chan**^2^** L. Kesavalu**^1,2,^ ***.

1. Department of Periodontology, College of Dentistry, University of Florida, Gainesville, FL 32610, USA; sjeepipalli@dental.ufl.edu (S.J.); parvathi.biotech@gmail.com; anarafaela.luz84@gmail.com; doc.colella@gmail.com; Sandhya.nadakuditi@gmail.com; desarajut@ufl.edu; ashithayada@ufl.edu; jonime@ufl.edu.
2. Department of Oral Biology, College of Dentistry, University of Florida, Gainesville, FL 32610, USA; echan@ufl.edu; JWilliams@dental.ufl.edu.
3. Department of Oral and Maxillofacial Diagnostic Sciences, College of Dentistry, University of Florida, Gainesville, FL 32610, USA; Ibhattacharyya@dental.ufl.edu.

***** Correspondence: kesavalu@dental.ufl.edu; Tel.: +1-352-273-6500

**Supplementary Material**


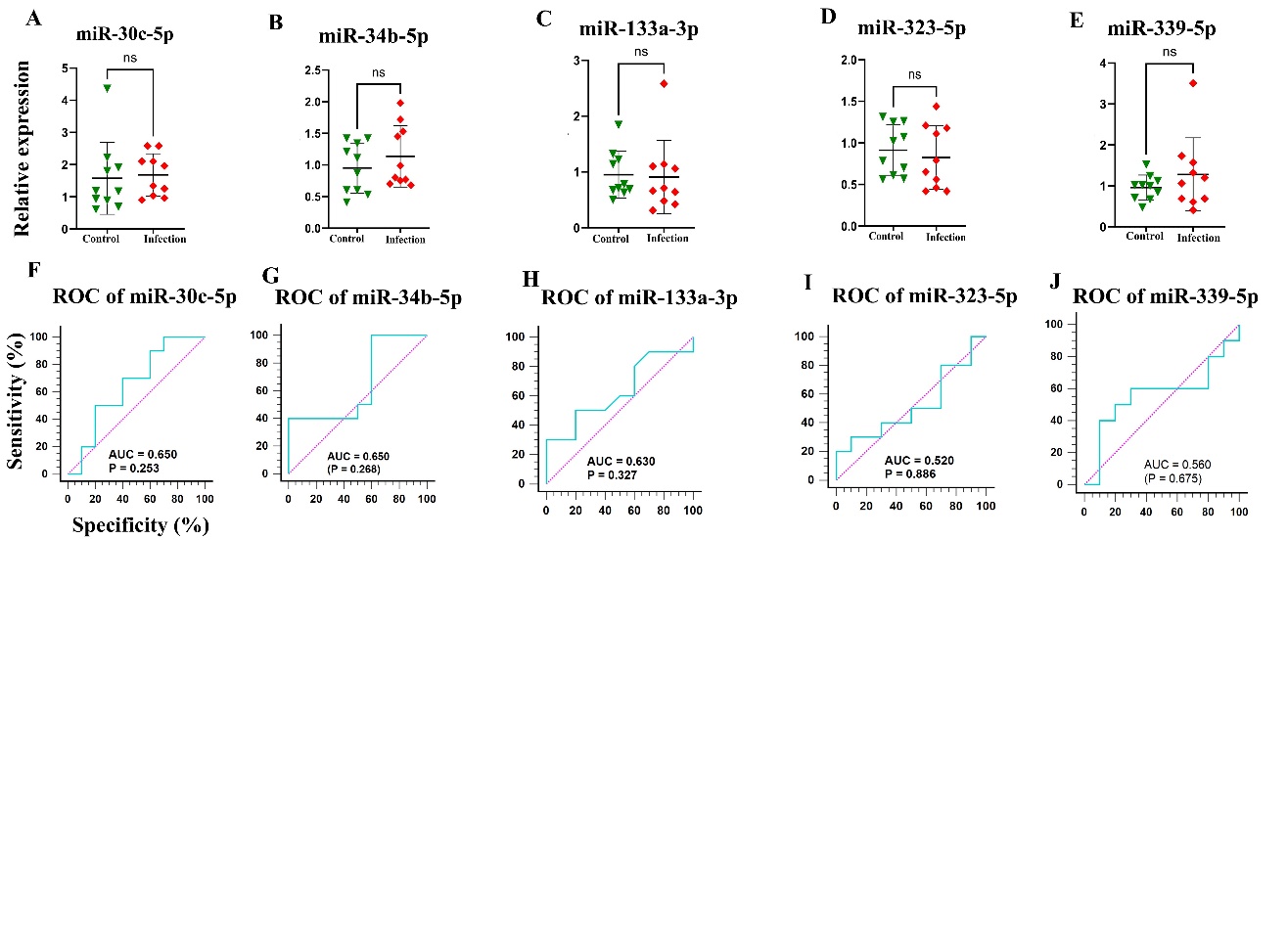


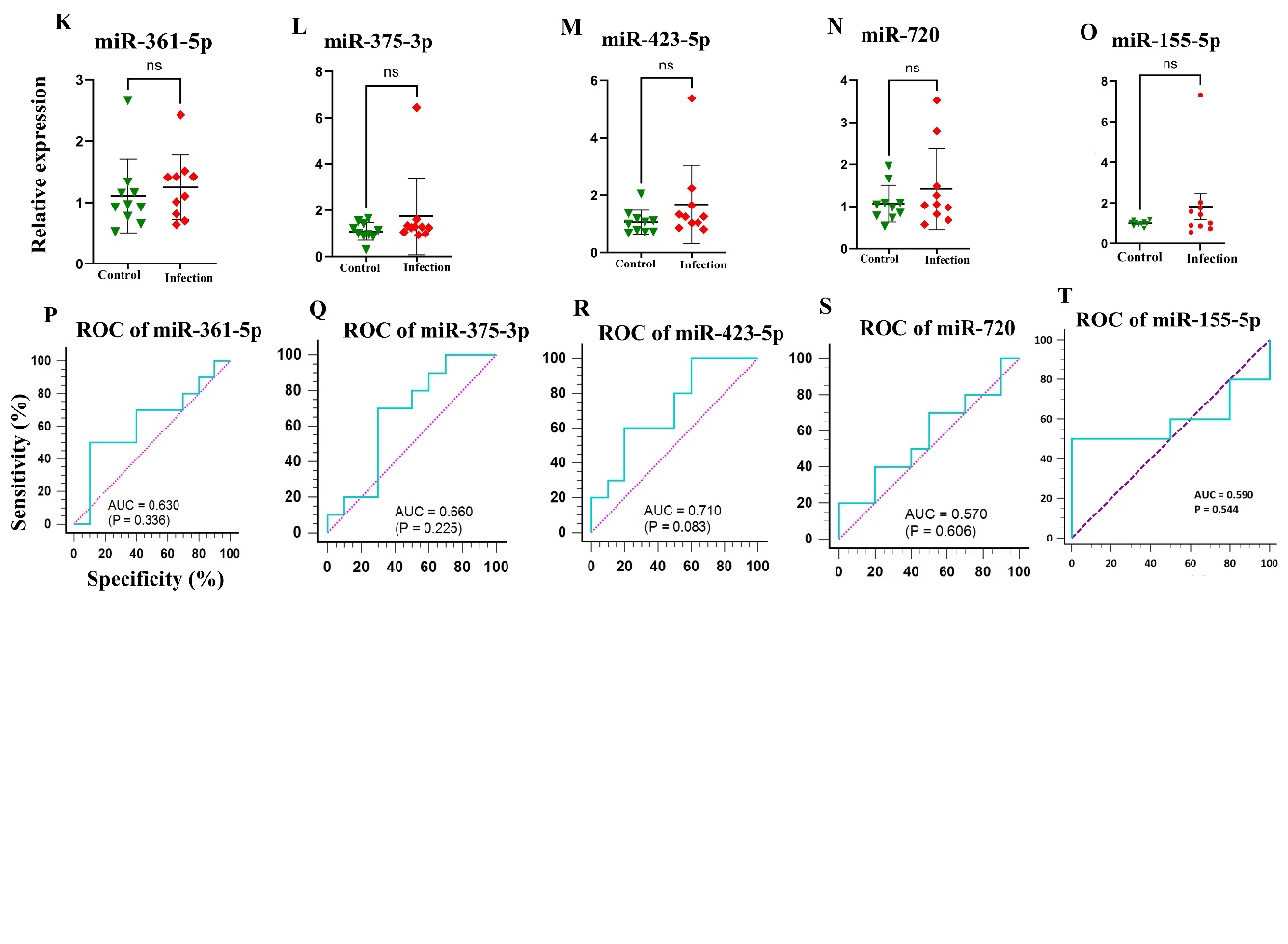


**Supplementary Figure S1.** Relative expression levels were determined by qRT-PCR for selected microRNAs (miRNAs) non-significant in the mandibles of the polymicrobial infected C57BL6/J wild-type and sham-infected mice. ROC curve of miRNAs that correlates with polybacterial infection-induced periodontitis.


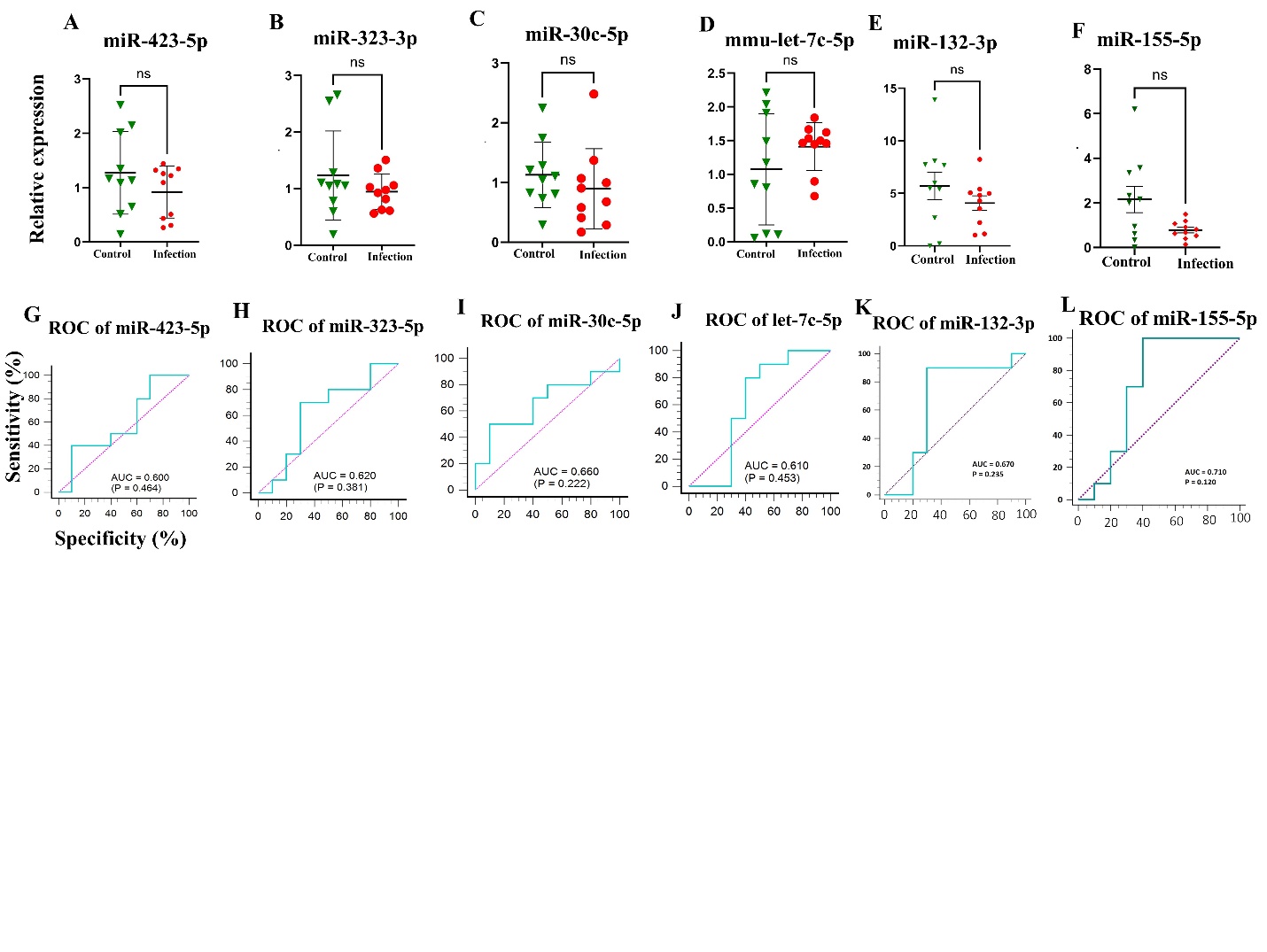


**Supplementary Figure S2.** Relative expression levels were determined by qRT-PCR for selected microRNAs (miRNAs) non-significant in the mandibles of the polymicrobial infected TLR2^-/-^ mice vs TLR2^-/-^ sham infected mice. ROC curve of miRNAs that correlates with polybacterial infection-induced periodontitis.


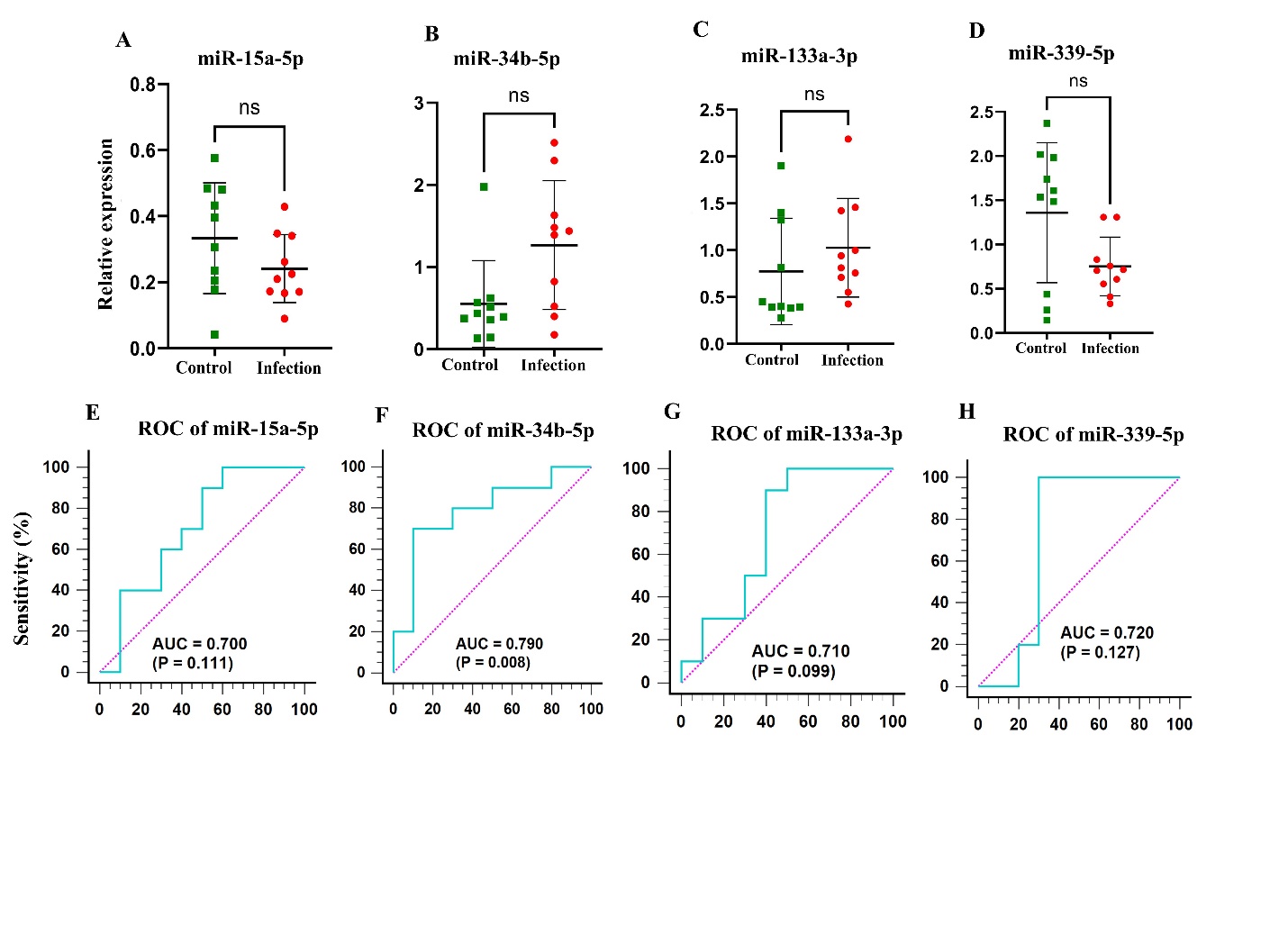


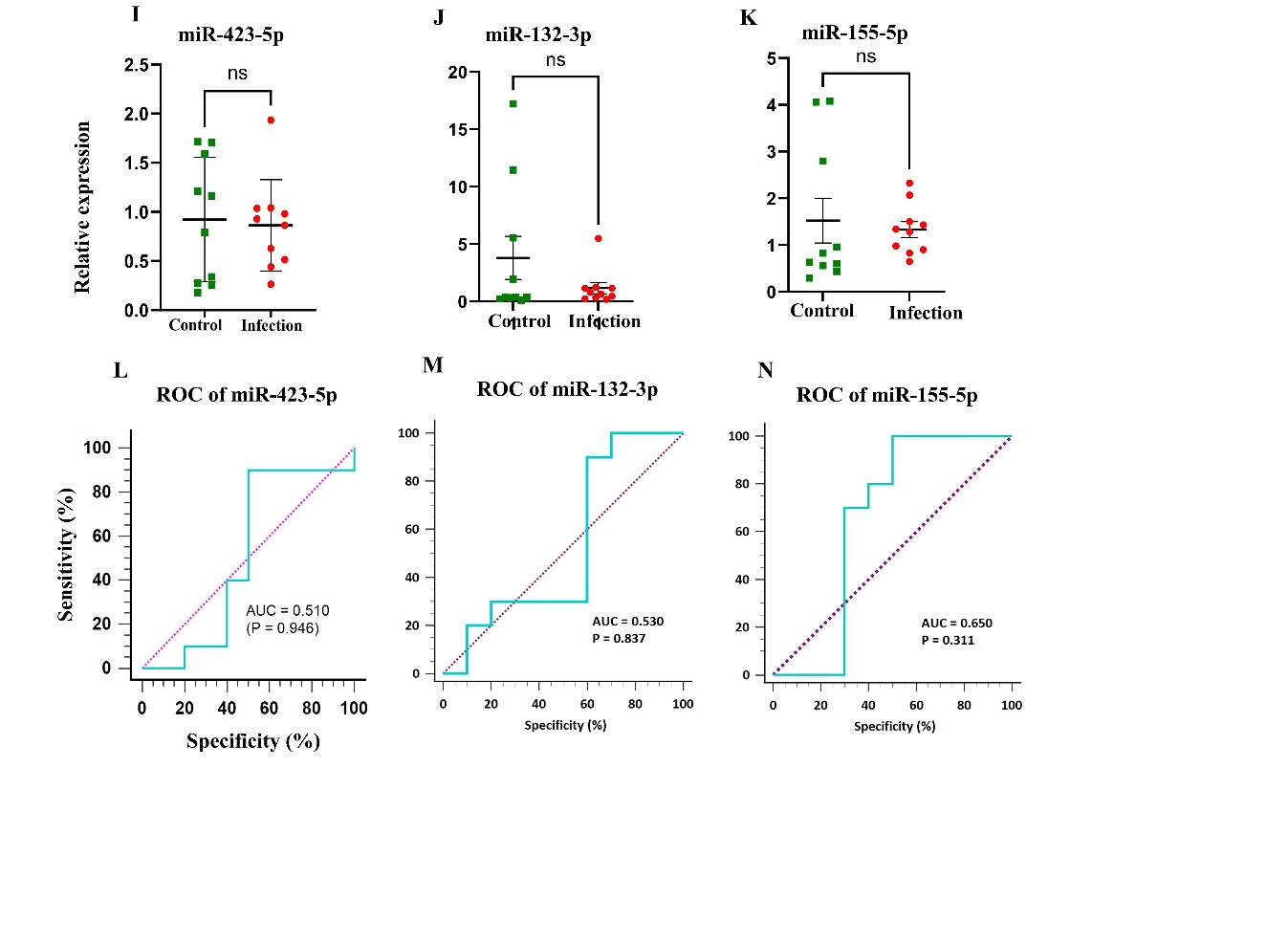


**Supplementary Figure S3.** Relative expression levels were determined by qRT-PCR for selected microRNAs (miRNAs) non-significant in the mandibles of the polymicrobial infected TLR4^-/-^ mice vs TLR4^-/-^ sham infected mice. ROC curve of miRNAs that correlates with polybacterial infection-induced periodontitis.
